# Supplementary material for: The Effect COVID Has Had on the Wants and Needs of Children in Terms of Play: Text Mining the Qualitative Response of the Happen Primary School Survey with 20,000 Children in Wales, UK between 2016 and 2021
Source: Int J Environ Res Public Health. 2022 Oct 4;19(19):12687. doi: 10.3390/ijerph191912687 (PMC9566363; doi:10.3390/ijerph191912687)
Supplement: Supplementary file 1 [file ijerph-19-12687-s001.zip › S2.pdf]

Supplementary file S2 (S2): Table S1

**Time To Play**
• *"Spending more time swimming and going outside"* (2016/2017)<br>• *"Everyone spends the time that they play devises to go for a walk at least"* (2017/2018)<br>• *"We should spend less time on our phones."* (2017/2018)<br>• *"Improve the park facilities so we can keep fit and have fun in our own time"* (2017/2018)<br>• *"Have more time to do sports"* (2018/2019)<br>• *"To have more clubs at lunch time break"* (2018/2019)<br>• *"I would probably change how much extra work we do after school so we can spend more time together."* (2018/2019)<br>• *"Not just boy sports at play times so girls can play aswell."* (2018/2019)<br>• *"Go spend time outside of the house"* (2019/2020)<br>• *"Allowed to play out all the time because we like playing out"* (2019/2020)<br>• *"More time to play"* (2019/2020)<br>• *"Less time on our electronics"* (2019/2020)<br>• *"I didn't have to homework because not being in schools is supposed to be my free time, doing school work at home i think is silly."* (2019/2020)<br>• *"More play time"* (2020/2021)<br>• *"To have time to have time to talk outside during lessons"* (2020/2021)<br>• *"Some more time to play at play centres and similer things like that."* (2020/2021)<br>• *"The amount of time i get to play with my friends"* (2020/2021)<br>• *"Afternoon play time"* (2020/2021)<br>• *"To have more time to play"* (2021/2022)<br>• *"Limit tv and time on devices."* (2021/2022)<br>• *"A limit of screen time and more fresh air"* (2021/2022)<br>• *"More breaktimes"* (2021/2022)
**Space To Play (including access, safety and sustainability concerns in play spaces)**
• *"We could get a park nearer to our house."* (2016/2017)<br>• *"To have a pavement running from [A] to [B]"* (2016/2017)<br>• *"To move the busy road by my house"* (2016/2017)<br>• *"Litter and dog poo and speeding"* (2016/2017)<br>• *"More rubbish bins and less cars"* (2016/2017)<br>• *"Less rubbish and shattered glass"* (2016/2017)<br>• *"Have a park built"* (2017/2018)

• *"More parks for people to run or walk around in instead of just staying inside on their devices"* (2017/2018)<br>• *"Get a little park in the local area where people could walk to or cicle to or scoot to so a lot of people (children or kids) could make frends and could hang out"* (2017/2018)<br>• *"People could start picking up their rubbish and put in the bin"* (2017/2018)<br>• *"Have a litter pick up once a week,raise more money for charity's,clean up dog poop after the dog poop's,build homes for headghogs and other animals,rent a big boat and go into the middle of the ocean and have a big litter pick for a week."* (2017/2018)<br>• *"Put more bins so that there is less litter."* (2017/2018)<br>• *"Longer breaks and be calmer and nicer with us"* (2018/2019)<br>• *"Get involved in more outdoor activities"* (2018/2019)<br>• *"To help school have better sporting facilities."* (2018/2019)<br>• *"There are unfriendly people hanging around my area doing drugs and smoking"* (2018/2019)<br>• *"Make sure that parks and other places have safe equipment"* (2018/2019)<br>• *"Play an the grass more in the summer"* (2019/2020)<br>• *"Make the park safer from unkind boys"* (2019/2020)<br>• *"More space to play outside"* (2020/2021)<br>• *"Make the yard bigger"* (2020/2021)<br>• *"I would like for new gates where the parking lot is and some fences where the cars can go to the parking lot so it's more safer and people won't worry"* (2020/2021)<br>• *"Easier to access out door places"* (2021/2022)<br>• *"Bigger park"* (2021/2022)<br>• *"A bigger Garden."* (2021/2022)<br>• *"More safer and clean areas for children to play and feel more comfortable!"* (2021/2022)
**Permission to Play (including relationships)**
• *"More friends to live by my house"* (2016/2017)<br>• *"Stop bullies"* (2016/2017)<br>• *"Do more sport with my mum"* (2016/2017)<br>• *"People not smoking as much and less fighting between people."* (2017/2018)<br>• *"Be very kind"* (2017/2018)<br>• *"Friends living more near"* (2017/2018)<br>• *"Stop fighting"* (2018/2019)<br>• *"Mum and dad to live with each other"* (2018/2019)<br>• *"My friends aka 1 or 2 people"* (2018/2019)<br>• *"Stop bullying"* (2019/2020)

• *"To have less arguments" (2019/2020)*<br>• *"Being around friends more" (2019/2020)*<br>• *"Going out with my friends" (2020/2021)*<br>• *"Not getting along" (2020/2021)*<br>• *"Maybe my some of my friends could be a bit nicer/kinder" (2020/2021)*<br>• *"Longer breaks and be calmer and nicer with us" (2020/2021)*<br>• *"Be kind to people and make others to be kind to others" (2021/2022)*<br>• *"Stop bullying around the world" (2021/2022)*<br>• *"To have my mum not be so busy" (2021/2022)*
**Specific Activity Recommendations**
• *"Add somewhere like a field or playground nearer to where people live so little children or even us can exercise as some children cant go that far. everyone should have fun exercising." (2016/2017)*<br>• *"Go swimming more" (2016/2017)*<br>• *"Swimming" (2016/2017)*<br>• *"Bike track" (2017/2018)*<br>• *"An all sports club"(2017/2018)*<br>• *"Having more exercise units such as football grounds." (2017/2018)*<br>• *"Play more games" (2018/2019)*<br>• *"I will change do more sports" (2018/2019)*<br>• *"More sport clubs" (2019/2020)*<br>• *"More PE" (2019/2020)*<br>• *"Swings in the park, better football pitch, somewhere to play basketball" (2019/2020)*<br>• *"More activities to do outside." (2020/2021)*<br>• *"Better playing equipment in the park for older children" (2020/2021)*<br>• *"To run with my friends more and play different activities." (2020/2021)*<br>• *"More football and breaks" (2021/2022)*<br>• *"More after school sports" (2021/2022)*<br>• *"More gym and physical activity" (2021/2022)*
**Health and Wellbeing Outcomes**
• *"Eat more fruit" (2018/2019)*<br>• *"To bring healthy snacks to school at first playtime." (2018/2019)*<br>• *"Sometimes bring around healthy fruit and veg" (2018/2019)*<br>• *"More fruit and veg" (2019/2020)*

- *"Learning things that are most important to the world and events" (2019/2020)*
- *"Keep fit always get fresh air" (2020/2021)*
- *"I think we should have things to play with and we need more fruits around us." (2020/2021)*
- *"To Do More Exercise" (2020/2021)*
- *"Try to cut down on sugar." (2021/2022)*
- *"If school did heifer diners" (2021/2022)*
- *"Have healthy food for healthy eating and more activities for plays" (2021/2022)*

The Impact of Covid

- *"i would change covid-19 so me and my friends can meet outside of school" (2019/2020)*
- *"Covid not being here" (2019/2020)*
- *"NO covid rules." (2019/2020)*
- *"Try and stop coved from spreading everywhere" (2021/2022)*
- *"Stop coronavirus and be able to be with people in partys more often." (2021/2022)*
- *"Get rid of COVID rules" (2021/2022)*
- *"seeing each other a lot more but because of covid we have not been doing that" (2021/2022)*
- *"Easier interaction not staying away from eachother (covid)" (2021/2022)*
